# Attentional bias retraining in cigarette smokers attempting smoking cessation (ARTS): Study protocol for a double blind randomised controlled trial

**DOI:** 10.1186/1471-2458-13-1176

**Published:** 2013-12-13

**Authors:** Rachna Begh, Marcus R Munafò, Saul Shiffman, Stuart G Ferguson, Linda Nichols, Mohammed A Mohammed, Roger L Holder, Stephen Sutton, Paul Aveyard

**Affiliations:** 1 Department of Primary Care Health Sciences, Radcliffe Observatory Quarter, UK Centre for Tobacco and Alcohol Studies, University of Oxford, Woodstock Road, Oxford OX2 6GG, UK; 2 Primary Care Clinical Sciences, University of Birmingham, Birmingham B15 2TT, UK; 3 UK Centre for Tobacco and Alcohol Studies, School of Experimental Psychology, University of Bristol, Bristol, UK; 4 MRC Integrative Epidemiology Unit (IEU), University of Bristol, Bristol, UK; 5 Department of Psychology, University of Pittsburgh, Pittsburgh, USA; 6 Schools of Medicine & Pharmacy, University of Tasmania, Tasmania, Australia; 7 School of Health Studies, University of Bradford, Bradford, UK; 8 Behavioural Science Group, Institute of Public Health, University of Cambridge, Cambridge, UK

## Abstract

**Background:**

Smokers attend preferentially to cigarettes and other smoking-related cues in the environment, in what is known as an attentional bias. There is evidence that attentional bias may contribute to craving and failure to stop smoking. Attentional retraining procedures have been used in laboratory studies to train smokers to reduce attentional bias, although these procedures have not been applied in smoking cessation programmes. This trial will examine the efficacy of multiple sessions of attentional retraining on attentional bias, craving, and abstinence in smokers attempting cessation.

**Methods/Design:**

This is a double-blind randomised controlled trial. Adult smokers attending a 7-session weekly stop smoking clinic will be randomised to either a modified visual probe task with attentional retraining or placebo training. Training will start 1 week prior to quit day and be given weekly for 5 sessions. Both groups will receive 21 mg transdermal nicotine patches for 8–12 weeks and withdrawal-orientated behavioural support for 7 sessions. Primary outcome measures are the change in attentional bias reaction time and urge to smoke on the Mood and Physical Symptoms Scale at 4 weeks post-quit. Secondary outcome measures include differences in withdrawal, time to first lapse and prolonged abstinence at 4 weeks post-quit, which will be biochemically validated at each clinic visit. Follow-up will take place at 8 weeks, 3 months and 6 months post-quit.

**Discussion:**

This is the first randomised controlled trial of attentional retraining in smokers attempting cessation. This trial could provide proof of principle for a treatment aimed at a fundamental cause of addiction.

**Trial registration:**

Current Controlled Trials: ISRCTN54375405.

## Background

Although many people who smoke achieve short-term success with current smoking cessation interventions, the rate of relapse to smoking remains high. Over 75% of initially successful quitters return to smoking within a year, with relapse occurring most commonly in the first 6 months after cessation [1]. At present, there is insufficient evidence to support the use of behavioural methods to prevent relapse in individuals achieving initial abstinence [2]. Most interventions have typically focused on Marlatt and Gordon’s [3] ‘skills’ based approach, which attempts to teach patients to identify situations conducive to relapse and teach cognitive and behavioural coping skills to deal with these situations. However, there is no evidence that the skills based approach diminishes or delays relapse to smoking [4] and so new interventions are required.

More recently, there has been increasing interest in the influence of implicit cognitive processing biases on our understanding of the relapse process [5]. Attentional bias, where drug users show excessive attention towards drug-related cues in the environment, is well-documented in tobacco smokers and users of other drug substances [6,7]. Several theoretical models of attentional bias propose that through repeated drug use, drug-related cues appear appealing to drug users, ‘grab’ attention and become ‘wanted’ to the extent that behaviour is guided towards drug use relatively automatically [8,9]. Further theoretical models propose that increases in attentional bias are both a cause and consequence of high levels of craving [10]. Thus, smokers with high levels of craving may be more likely to search the environment for smoking-related cues, while prolonged attentional processing of smoking-related cues could increase urges to smoke. There is evidence that attentional bias is related to drug craving [11], although this relationship has been reported as somewhat weak [12]. Attentional bias has also been linked to an increase in the likelihood of relapse in smokers [13], alcohol users [14] and heroin addicts [15]. There is also strong anecdotal evidence that environmental and situational cues act as triggers for relapse in abstinent smokers. Control of attentional processes may therefore play a key role in preventing relapse among smokers who are attempting to quit, although current smoking cessation programmes delivered by the National Health Service (NHS) Stop Smoking Services (SSS) in the United Kingdom (UK) do not target attentional biases directly.

Several paradigms measure cognitive processing biases. The most common among these are the visual probe task [16] and Stroop task [17]. In the standard version of the visual probe task, pairs of pictures or words – one drug-related and one neutral stimulus - are presented briefly on a computer screen before a probe appears in the location formerly occupied by one of the pictures or words. Smokers, in comparison to non-smokers, have shown an attentional bias towards smoking by responding faster to probes that appear in the location of smoking-related stimuli rather than neutral stimuli [18,19]. Other drug users have shown an attentional bias towards drug-related stimuli of their choice; for example, cannabis users are faster to respond to cannabis-related stimuli than neutral stimuli [20]. Similarly, the addiction Stroop task has been used extensively to measure selective processing of drug-related stimuli [21]. Processing biases on the Stroop task have been demonstrated in smokers who are typically slower to colour-name smoking-related stimuli than neutral stimuli [22,23].

There is some evidence to suggest that cognitive processing biases are apparent in smokers ever after attempts to stop smoking. The first study to explore this found that ex-smokers, who were enrolled in a smoking cessation programme and had been abstinent from smoking for at least 1 week, had an intermediate bias for smoking-related stimuli, in-between that of smokers and non-smokers [19]. In another study, smokers who were attempting to quit and smokers without such plans had similar levels of attentional bias [24]. In contrast to these studies, two studies found that ex-smokers showed a similar level of processing bias as never-smokers, while smokers exhibited more bias than both other groups [23,25]. In both of these studies, ex-smokers had been abstinent for at least 6 months rather than recently abstinent from smoking. Thus, data are conflicting but it is possible that attentional bias persists early in a quit attempt and resolves with increased duration of abstinence although no study has examined this. However, if attentional bias persists for many months, we might speculate that if there are enduring effects of smoking cues after cessation as suggested in previous research, abstinence could be undermined in initially successful quitters.

Cognitive bias modification (CBM) procedures have been developed in an attempt to change cognitive processing biases using modified versions of the visual probe task and Stroop task [26-33]. CBM procedures are designed to augment or attenuate cognitive processing biases, providing not only a method of studying the causal relationship between attentional bias, craving and relapse, but also a potential treatment for attentional bias in clinical populations. Attentional retraining (AR) is most commonly used in the study of addiction-related attentional bias. Individuals with specific drug use patterns can be trained to increase or decrease attentional bias towards their drug of choice in train-to-attend and train-to-avoid manipulations, respectively [26,28-30,32]. In the train-to-attend manipulation, probes replace drug-related stimuli more frequently than neutral stimuli; by comparison in the train-to-avoid manipulation, probes replace neutral stimuli on a greater number of occasions. Consequently attention is trained towards one particular stimulus type.

Most investigations of AR in addiction have been conducted in laboratory studies of non-treatment seeking alcohol users [28,29,32] and more recently, tobacco smokers [26,30,31]. These studies have typically measured the effects of AR on subjective outcomes, e.g. craving and behavioural outcomes such as drug consumption as well as the change in attentional bias. In the first of these studies comparing a train-to-attend with a train-to-avoid manipulation, training towards alcohol-related stimuli was associated with increases in attentional bias, urge to drink and actual beer consumption. Conversely, training away from alcohol-related stimuli was associated with reductions in attentional bias and beer consumption but not urge to drink. The study was replicated with the inclusion of a control group that received no training towards any particular stimulus type; the predicted direction of change in attentional bias was observed in the groups trained to attend or avoid alcohol-related stimuli, as well as no change in attentional bias in the control group [29]. Alcohol craving increased among participants in the attend group, but only for those who were aware of the experimental contingencies, i.e. participants who reported the relationship between the location of the probe and stimulus-presentation correctly in a post-task questionnaire. However, in contrast to the findings of the earlier study, there were no differences between groups in the volume of beer consumed. Schoenmakers and colleagues carried out a similar study where heavy social drinkers had learned to avoid alcohol-related stimuli and developed an attentional bias towards soft drinks, although training had no effect on craving or drink choice [32].

Only three laboratory studies have published findings on AR procedures in current tobacco smokers [26,30,31]. The first of these studies found that AR increased attentional bias in participants who were trained towards smoking-related stimuli and decreased attentional bias in those trained towards neutral stimuli [26]. Furthermore, when participants were measured on their response to a lit cigarette following the training procedure, greater increases in subjective craving were found in male participants who attended to smoking-related stimuli than those trained towards neutral stimuli. However, no effect of training on smoking topography (e.g. number of puffs taken, puff duration, etc.) was observed. In a replication of the study with a no-training control group, attentional bias was greater after training in the attend group than the avoid and no-training control groups, but these effects disappeared after 1 day [30]. Neither the train-to-attend or train-to-avoid manipulations had any effect on urge to smoke, although unlike in the previous study, no cue exposure task was used. No group effects of retraining on motivation to smoke were observed. McHugh and colleagues compared an avoid group with a no-training control group and found no change in attentional bias and no effects of retraining on subjective craving [31]. Unlike the two previous studies, no behavioural measures of tobacco-seeking were taken.

Collectively, laboratory studies of AR suggest that attentional bias can be modified and that, in some cases, corresponding changes in craving occur. While AR has not shown any effects on drug-taking behaviour in smokers, it is worth noting that laboratory studies of AR typically recruit samples of continuing smokers who are temporarily abstinent for the purpose of the experimental investigation. These smokers presumably have no motivation or intention to reduce their substance use in comparison to treatment-seeking smokers. Studies of AR in clinical populations are capable of addressing how attentional bias relates to real-world behaviour – particularly relapse - in addition to assessing the efficacy of AR as a clinical intervention.

Only two studies to date have examined the effects of AR in substance users seeking to reduce or abstain from drug use [27,33]. In an uncontrolled trial of AR, hazardous and harmful drinkers interested in reducing their alcohol intake completed 2 or 4 weekly sessions of AR on a modified Stroop task, respectively [27]. After treatment was complete, processing biases towards alcohol-related stimuli reduced in both groups, as did alcohol consumption by approximately 10 Units/week (1 Unit is equivalent to 8 g of ethanol) for the harmful drinkers. These reductions were also maintained at the 3 month follow up. Uncontrolled trials in people seeking to change their behaviour are difficult to interpret, however. In the only randomised controlled trial of AR in substance users, Schoenmakers and colleagues found that alcohol-dependent patients were more able to disengage attention from alcohol-related stimuli than control patients after 5 sessions of AR on a modified visual probe task, given in addition to standard treatment [33]. Moreover, relapse was delayed by over a month in patients that received AR. While there appear to be promising effects of AR as a clinical intervention in alcohol abusers, little is known about the clinical value of AR procedures in smokers attempting to quit.

### Rationale for the trial

Resumption of smoking by initially successful quitters is arguably the greatest public health challenge in smoking cessation. While there are few interventions at present that are known to reduce the risk of relapse to smoking [2], the development of new approaches like AR could be worthwhile. Despite evidence from laboratory studies indicating that attentional bias can be modified in tobacco smokers using AR procedures [26,30] and the success of such tasks on improving clinical outcomes in other addictions [33] and psychopathologies [34], no study has yet explored the clinical application of these procedures in treatment-seeking smokers.

We therefore propose a double-blind randomised controlled trial of multiple sessions of attentional bias retraining in smokers attempting smoking cessation (ARTS). This translational study offers the ability to both examine the benefits of AR on users of stop smoking services and provide findings to aid our understanding on the phenomenon of attentional bias and its relation to craving, withdrawal symptoms, lapses and relapse in smokers attempting to quit.

### Aims and study questions

The aim of the study is to investigate the efficacy of an AR intervention on attentional bias and smoking cessation outcomes in smokers undertaking behavioural treatment. The following study questions will be addressed:

1) Can AR diminish attentional bias in smokers during cessation; are the effects evident across different cognitive bias tasks and different types of stimuli?

We will investigate whether AR – using multiple sessions - can lead to reductions in attentional bias in smokers who are attempting cessation. If retraining is successful, participants should be able to demonstrate that they can divert their attention away from smoking cues on a visual probe task. We expect AR to reduce the degree to which smokers notice smoking cues in their environment because they are trained away from attending to them.

Similarly, if AR shows material reductions on one cognitive bias task, it is plausible that a reduction may be seen on another task measure - such as the pictorial Stroop task - if similar attentional processes are involved. Finally, if AR produces a global change in attentional bias and not just a task-specific change in bias towards smoking cues, then smokers should be able to transfer their ability to divert their attention away from other smoking cues that are not featured in the retraining procedure.

2) Does AR affect urges to smoke, cue-induced craving or withdrawal symptoms in smokers during cessation?

We will investigate the effects of AR on urges to smoke, cue-induced craving and withdrawal symptoms in smokers during their cessation attempt. If AR procedures are capable of reducing exposure by diverting attention away from smoking cues, this in turn could reduce the capacity of these cues to invoke craving and symptoms of withdrawal.

3) Do the effects of AR on attentional bias persist up to 6 months after cessation?

One marker for the success of AR procedures is to evaluate whether they produce enduring effects; this is particularly pertinent if the presence of attentional bias undermines abstinence [35]. As the durability of AR remains unclear at present, we will investigate whether the effects of AR are evident in smokers after their cessation attempt at follow-up assessments.

4) Does AR reduce the likelihood of relapse in smokers attempting cessation?

We will assess whether retraining can reduce the likelihood of relapse in smokers attempting to quit. If the ability to divert attention away from smoking-related cues during retraining translated to a smoker’s natural environment, s/he might experience less exposure to the environmental cues that would normally trigger smoking; in time, this could weaken the stimulus–response association between smoking cues and smoking behaviour, thus reducing the likelihood of a lapse occurring. Alternatively, if attentional avoidance leads to less instances of craving, this may also in turn reduce the likelihood of relapse, given that craving predicts relapse [13,36,37].

## Methods/design

This is a double blind randomised controlled trial. Participants attending a 7-session weekly NHS stop smoking clinic will be individually randomised to either an intervention group consisting of a modified visual probe task with AR or a control group with placebo training (PT). Five sessions of AR or PT will be delivered. Both groups will receive nicotine replacement therapy (NRT) in the form of transdermal nicotine patches and standard withdrawal orientated behavioural support [38].

### Inclusion criteria

Participants will be required to meet the following inclusion criteria to be eligible for enrolment into the trial:

1. Aged 18 years or over.

2. Currently smoke at least 10 cigarettes per day or 12.5 grams of tobacco or have a value of at least 10 parts per million (ppm) for exhaled carbon monoxide (CO).

3. Have normal or corrected-to-normal vision.

4. Informed consent.

5. Are able and willing to complete all study procedures.

### Exclusion criteria

Participants will be excluded if they present with any of the following:

1. A medical condition that prevents them from seeing the computerised images properly, attending to the task, or pressing the keyboard buttons on the computer accurately, or completing any other study procedures.

2. Are currently using nicotine replacement therapy (NRT), bupropion, nortriptyline, mecamylamine, reserpine, or varenicline, or undergoing any treatment for tobacco dependence (e.g. acupuncture) that they are not willing to cease using and instead use study medication.

3. Have previously had severe skin reactions to nicotine patches or severe eczema or other skin diseases that make patch use hazardous or undesirable.

4. Have a severe acute or chronic medical or psychiatric condition or previously diagnosed clinically important renal or hepatic disease, which could increase the risk associated with study participation or could interfere with the interpretation of study results and, in the judgment of the investigator, would make the participant inappropriate for entry into this study.

### Withdrawal criteria

It is standard practice in smoking cessation trials to treat those who fail to attend appointments as having relapsed [39]. Therefore, failure to attend will not be defined as withdrawal from the trial; we consider that the only withdrawals will be those in which the participant has asked to be withdrawn. We expect this in less than 5% of participants. This is standard procedure in smoking cessation studies.

### Participant recruitment

**Figure 1:**
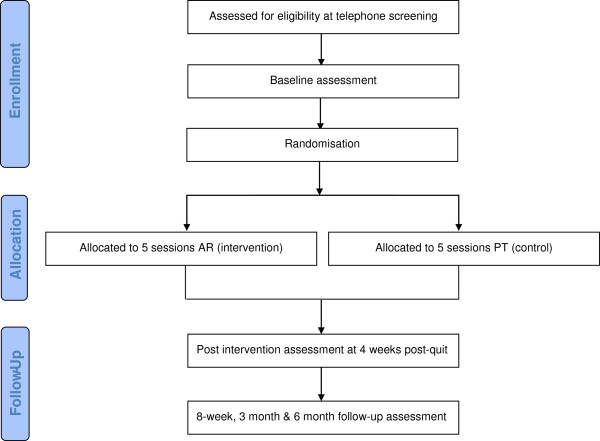
Flow diagram of participants through trial.

Figure 1 shows the flow of participants through the trial. Participants will be recruited from West Midlands NHS SSS. A letter of invitation and a patient information sheet about the study will be sent from GP practices to patients that are registered on their databases as smokers. The letters will ask those patients who wish to take part in the trial to respond to the study team. In our experience, we anticipate that 5–10% will respond. Staff within the NHS SSS will also write to smokers with a history of failed quit attempts who are on their databases. Preliminary eligibility to participate will be assessed during telephone screening and potential participants will be booked in for an assessment session at the clinic site, similar to that arranged by the NHS SSS. Written informed consent will be obtained from all participants at the first session, which takes place 2 weeks prior to quit day.

### Staff training

Research nurses and stop smoking advisors (SSAs) will be trained to deliver the intervention. All staff will complete a 2-day NHS stop smoking advisor course. They will also attend a training day in which they will be briefed on the clinical procedure on how to deliver each task and on use of the trial database. Prior to running a clinic, each member of staff will observe a baseline session and week −1 (randomisation) session delivered by the chief investigator. In turn, the chief investigator will observe the first two sessions delivered by each nurse/SSA involved in the study. Regular site visits will be conducted to check that the intervention is being delivered as per protocol.

### Trial procedures

Participants in both trial arms will be seen weekly in clinics from 2 weeks prior to quit day up to 4 weeks post quit day. There are ten clinic sessions in total. Randomisation takes place at the second clinic session, which initiates the first of the 5 weekly AR/PT sessions. Follow up visits take place at 8 weeks and 3 months post quit day, with a final visit arranged at 6 months. Participants will be paid £15 to complete assessments at 3 month and 6 month follow-up sessions, as these are not therapeutic encounters. Participants will be reminded to attend their appointments by telephone or text message. Staff will complete a case report form (CRF) at each clinic visit, which contains a checklist of the trial procedures.

### Randomisation

Participants will be randomised 1:1 to either AR or PT using a computer generated simple randomisation scheme, ordered in random permuted blocks of four. The sequence was generated by the trial statistician and entered on to a dedicated online trial database by an independent programmer in the Primary Care Clinical Research and Trials Unit (PCCRTU) at the University of Birmingham. At 1 week prior to quit day, at the start of the clinic session, the therapist will access the randomisation section of the trial database and click on a button that reveals a letter (‘A’ or ‘B’) to reveal the training task to which the participant is allocated. The training tasks are contained within two folders labelled ‘Training A’ or ‘Training B’ on the study laptop, which conceals whether the procedure is AR or PT. These folders were labelled by an independent researcher prior to the start of the trial. Thus the participants, therapists and study staff will be blinded to allocation, to minimize the risk of bias.

### Measures

Table 1 displays the treatment and measurement plan for the study. The measures consist of the following:

• A baseline questionnaire to collect information on the demographic and clinical characteristics of participants. Participant age, gender, ethnicity, education and employment status are classified using UK Census 2011 categories [40]. The questionnaire also contains information on smoking history including the Fagerström Test of Nicotine Dependence (FTND) [41], a 6-item measure assessing the severity of nicotine dependence.

• A visual probe task and pictorial Stroop task to assess attentional bias.

• The Mood and Physical Symptoms Scale (MPSS) [42]. This will be administered at the beginning of every session to assess urge to smoke and withdrawal. A modified version of the MPSS will be used in which each of the nine items is rated on a scale from 1–7. Items relating to the strength and frequency of urges can be combined to produce a composite score (MPSS-C); this is also the case for combined mood items (MPSS-M). The MPSS was preferred over other measures such as the Questionnaire of Smoking Urges because of its superiority in predicting treatment outcomes [43,44].

• Exhaled carbon monoxide (CO). Readings will be taken at the beginning of each session to biochemically verify smoking status.

• A visual analogue scale (VAS) to measure cue-induced craving at the beginning of the second session and following attentional bias assessments. Measurements will be recorded on a 100 mm scale from “Not At All” to “Extremely” prior to and after the task.

• Ecological Momentary Assessment (EMA) [45] to collect information on lapses. EMA is an approach to collecting data in real-time on hand-held electronic devices; it does not carry the risk of recall bias like paper diaries [46]. Participants will be given an electronic diary at the first session and they will be instructed to record any lapses that occur and the circumstances in which the lapse occurs up to 5 weeks post quit day. Those who use an electronic diary will be paid up to £75 at the 8-week session for completing assessments in this way.

• A questionnaire on knowledge of group allocation.

• A patient satisfaction questionnaire developed by the study team on the acceptability of the training tasks. Two items relate to how difficult the task is to understand and carry out, while a further two items assess the convenience of task. Items are rated on a 5-point scale ranging from “not at all difficult” to “extremely difficult” and “very convenient” to “very inconvenient”.

**Table 1:** Treatment and assessment schedule

**Session**	**Treatment**	**Measures**
Baseline (week −2)	Withdrawal-oriented behavioural support	• Baseline questionnaire [demographics, smoking history, Fagerstrom Test for Nicotine Dependence (FTND)]
		• Mood and Physical Symptoms Scale (MPSS)
		• Exhaled carbon monoxide (CO)
		• Attentional bias assessment (visual probe and Stroop tasks)
Pre-quit visit (week −1) Randomisation week	1 week supply nicotine patches Withdrawal-oriented behavioural support Intervention group receives attentional retraining (AR). Control group receives placebo training (PT)	• CO
		• MPSS
		• VAS measure of craving (pre & post cue exposure task)
Quit day (week 0)	1 week supply nicotine patches Withdrawal-oriented behavioural support Intervention group receives AR Control group receives PT	• CO
		• MPSS
Post-quit visits (weeks +1, +2, +3)	1 week supply nicotine patches Withdrawal-oriented behavioural support Intervention group receives AR. Control group receives PT	• CO
		• MPSS
		• Lapses recorded on electronic diary
Week +4 post-quit visit	4 week supply nicotine patches Withdrawal-oriented behavioural support	• CO
		• MPSS
		• Attentional bias assessment (visual probe and Stroop tasks)
		• VAS measure of craving (pre & post cue exposure task)
		• Group allocation assessment
		• Lapses recorded on electronic diary (up to +5 weeks) thereafter reported in clinic CRF
Week +8 post-quit visit	4 week supply transdermal nicotine patches (where eligible)	• CO
		• MPSS
		• Attentional bias assessment (visual probe and Stroop tasks)
		• VAS measure of craving (pre & post cue exposure task)
		• Lapses reported in clinic CRF
3 months post-quit visit		• CO
		• MPSS
		• Attentional bias assessment
		• (visual probe and Stroop tasks)
		• VAS measure of craving (pre & post cue exposure task)
		• Lapses reported in clinic CRF
6 months post-quit visit		• CO
		• MPSS
		• Attentional bias assessment (visual probe and pictorial Stroop tasks)
		• VAS measure of craving (pre & post cue exposure task)
		• Lapses reported in clinic CRF
		• Patient satisfaction questionnaire

### Materials

Eighteen picture pairs of smoking-related and matched neutral pictures will be used across attentional bias assessment and training tasks (picture pairs 1–18). These stimuli have been tested and applied in previous research [47,48]. Each set of pictures consists of a colour photograph of a smoking-related stimulus or scene (e.g. a close-up of a cigarette) matched on age, sex, complexity and ethnicity to another photograph containing no smoking-related content. In the assessment version of the visual probe task and pictorial Stroop task, 12 picture pairs will be used (picture pairs 1–12). Similarly in the AR and PT versions of the visual probe task, the 12 picture pairs consist of 6 picture pairs featured in the assessment version of the task (picture pairs 6–12) in addition to 6 new picture pairs (picture pairs 13–18). An extra 4 neutral picture pairs will be used for practice trials before each task.

### Clinic Tasks

• Visual probe task

• At the baseline session and again at 4 weeks post-quit, 8 weeks, 3 months and 6 months, all participants complete the assessment version of the visual probe task. The assessment version, which will be used to measure attentional bias, comprises a total of 192 trials, presented in two blocks. Each trial begins with a fixation cross displayed in the centre of the computer screen for 500 ms. A picture pair of smoking-related and neutral pictures is then presented side-by-side on the screen for 500 ms. After this picture pair disappears, a visual probe is presented in the location formerly occupied by one of the pictures. This probe is either a circle or square. Participants are required to discriminate the identity of the probe and respond accordingly by pressing the up or down arrow keys on the keyboard as quickly as possible. There is a 500 ms interval before the next trial. Presentation of each picture-pair and probe location is counterbalanced. In all trials, the visual probe replaces the smoking-related and neutral pictures with equal frequency. At the start of the task, participants carry out 8 practice trials in which neutral picture pairs are presented first, to allow them to become familiar with the procedure.

• Each block of trials is presented in a new random order for each participant, using EPrime version 2 (Psychology Software Tools Inc., Pittsburgh PA). The task takes approximately 16 minutes. Attentional bias scores will be calculated from reaction time (RT) data; an attentional bias towards smoking cues is characterized by faster reaction times towards smoking-related pictures than neutral pictures.

• Pictorial Stroop task

• All participants will carry out a pictorial Stroop task as an additional measure of cognitive bias. The pictorial Stroop task will be given after the visual probe task at the baseline session and again at 4 weeks post-quit, 8 weeks, 3 months and 6 months. The task comprises a total of 192 trials, presented in four blocks of 48 trials, with each block consisting of smoking-related pictures or neutral pictures only. Each picture is presented centrally on a computer screen with either an outline of a red, blue, yellow or green border. Participants are required to indicate the colour of the border, while ignoring the picture, by pressing one of four corresponding labelled keys on the keyboard, as quickly as possible. Participants receive 8 practice trials in which neutral pictures are presented first, to allow them to become familiar with the procedure. A short break between blocks will be permitted.

• Each block of trials is presented in a new random order for each participant, using EPrime version 2 (Psychology Software Tools Inc., Pittsburgh PA). The task takes approximately 12 minutes to complete. Stroop bias scores will be calculated from RT data; selective processing of smoking cues is characterized by slower reaction times towards smoking-related pictures than neutral pictures.

• Cue exposure task

• At 1 week prior to quit day, 4 weeks post-quit and follow-up sessions at 8 weeks, 3 months and 6 months, participants in both groups will be given a cue exposure procedure to measure cue-induced craving immediately after completion of the visual probe task and pictorial Stroop task. This is a common procedure in cue-reactivity research [49,50]. Showing a strong craving response to cue-exposure has been shown to predict relapse risk [51]. Prior to attending the session at week −1, participants will be instructed to abstain from smoking for at least 1 hour. We chose an abstinence period of 1 hour to avoid floor effects in craving ratings, commonly found immediately after smoking [52].

• In order to standardize the procedure, instructions for the cue exposure task will be recorded on a digital recorder and then played to participants in the relevant clinic sessions. Before the instructions are played, participants will provide a single rating of their urge to smoke on the VAS. The therapist will place a box that conceals a cigarette and a lighter in front of the participant. The recording will then be played, which instructs the participant to lift up the box and handle the cigarette and lighter contained within. The task lasts 3 minutes. Following the task, participants will provide another rating of their urge to smoke on the VAS.

### Control group

Participants allocated to the control group will carry out 5 weekly sessions of PT, starting 1 week prior to their designated quit day. During each session, participants perform 8 practice trials of neutral picture pairs followed by 192 trials of PT, presented in a block of two. Between each block, participants are permitted to have a short break if required. The task takes approximately 16 minutes to complete. On each PT trial, the visual probes always replace smoking-related and neutral pictures with equal frequency.

### Intervention group

Participants allocated to the AR group will carry out 5 weekly sessions of the modified visual probe task, AR, starting 1 week prior to their designated quit day. Eight practice trials of neutral picture pairs are presented prior to the first block of AR trials. A total of 192 training trials are presented in a block of two, where participants have the opportunity to have a break in between. The task takes approximately 16 minutes to complete.

The AR program differs from the PT program only in the location of the visual probes. During each training trial, visual probes always appear in the location of the neutral pictures. Thus, participants always have their attention directed away from smoking-related pictures.

### Stop smoking treatment

Systematic reviews have shown that some behavioural and pharmacological interventions increase people’s chances of successfully stopping smoking [53,54]. All participants will therefore be given NRT and receive standard withdrawal orientated behavioural support [38].

Participants in this trial will be offered 21 mg/24 hour nicotine patches as the only choice of treatment. This is because:

1) All participants will be regular smokers for whom the 21 mg dose is deemed appropriate.

2) The study aims to examine the effects of AR on urge to smoke. Short-acting NRT e.g. inhalator or gum affect cue-induced urges to smoke and reduce their intensity [55]. It would thus be difficult to assess the effects of the AR if short-acting NRT is used. Participants are also not permitted to use varenicline for the same reason [56]. Investigators have found that nicotine patches do not protect against cued craving [57], therefore we consider that patch-use is unlikely to mask the potential effects of retraining.

3) The patch is the best tolerated form of NRT and has the highest adherence [58,59].

### Dose alteration procedure

Nicotine patches are well tolerated in the large majority of regular smokers and so we expect that most people will continue with the standard dose. However, there are circumstances when the form or dose of the preparation needs to be changed. This variation reflects pragmatic behaviour in the NHS SSS and is expected to be equal in both arms. We anticipate the following occurrences:

1) Minor skin irritation to the patch is one of the most common problems with use. This is commonly eased by swapping from one form of patch to another, because it is usually intolerance to the glue. If the skin reaction is worse, such as causing blisters that cannot be remedied by emollients and hydrocortisone cream, patch use will be stopped and the participant will be swapped to an equivalent dose of oral NRT.

2) Sleep disturbance or vivid dreaming is also one of the most common problems with use of the nicotine patch. This can usually be eased by removing the nicotine patch an hour or so before bedtime and so this will be advised. There is no good evidence that 16 hour patch use is less effective than 24 hour patch use.

3) Possible symptoms that dose is too high are uncommon problems, but possible. Nausea is the earliest symptom of overdose, but it is also a common symptom experienced by people often enough. Nicotine has a short half life, meaning that by about 10 hours after first applying a patch, nicotine has reached a steady state. Therefore nausea occurring for the first time days after starting treatment is unlikely to be due to the patch. More definite symptoms are as follows, muscular twitching, dizziness, confusion, rapid pounding heart, high blood pressure, vomiting, and weakness. However, 21 mg/24 hour patch systems come as 14 mg/24 hours and 7 mg/24 hours, which can be used in a step down system. If the therapist thinks that an overdose is likely, the precaution will be to step down the dose to the next step i.e. from 21 mg to 14 mg, or from 14 mg to 7 mg.

### Duration of treatment and instructions for use

Treatment with NRT will start either on the evening prior to quit day or the morning of quit day, depending on personal preference. Patches will be dispensed accordingly during the second visit, which is 1 week prior to quit day. Instructions for patch use include changing it every 24 hours, using a different area of skin for the new patch. Participants will be advised to continue using the patch for at least 8 weeks or stop if they abandon their quit attempt before the 8 weeks. The therapist in consultation with the patient may choose to step down the patch as discussed above. Step down is not necessary as there is no evidence to suggest that it enhances efficacy, but it is commonly perceived as helpful by patients. Step down towards the end of treatment will not be permitted to commence until at least 4 weeks after quit day. The therapist will be instructed not to suggest stepping down in people who have had recent lapses. Some organisations we are working with do not allow treatment for longer than 8 weeks, but, in those that do, the therapist should consult the participant about longer courses of treatment up to 12 weeks duration. This decision will be at the discretion of the therapist in consultation with the patient.

Behavioural support will start 2 weeks prior to quit day, and last up to 4 weeks after quit day. This follows the typical 7-session withdrawal orientated therapy programme offered in existing NHS SSS [38].

### Reporting of adverse events

This is not a trial of an investigational medical product. We are using a licensed medical product within the terms of its license and in accord with clinical guidelines. We therefore expect relatively few problems and so there are no special reporting requirements. The therapist leading the sessions will manage problems within his/her own competence. Clinical advice will be sought from the trial doctor. Between them, the therapist and trial doctor will decide how to manage unexpected problems and whether to report a suspected unexpected serious adverse reaction (SUSAR) to the Medicines and Health Care Regulatory Authority using the yellow card system (this is a standard system for reporting unusual reactions to medication).

However, for the purposes of the trial, we will record clinically significant adverse events that lead to a change in medication management or is considered to be significant otherwise. This will allow us to track changes in medication instruction, such as swapping to 16 hour use or dose alterations. The CRF will be used to record the date, the nature of the adverse event/symptoms, and the action taken.

### Primary trial outcomes

• Measure of attentional bias during assessment trials of the visual probe task, as measured by the difference in median reaction time (ms) taken to respond to probes replacing smoking-related stimuli versus probes replacing neutral stimuli. This will be assessed at 4 weeks post-quit in abstinent and non-abstinent smokers across both trial arms, following recommendations of Shiffman et al. [60].

• Strength of weekly urge to smoke on the MPSS, measured up to 4 weeks post-quit in abstinent and non-abstinent smokers across both trial arms.

### Secondary trial outcomes

• Strength of weekly withdrawal symptoms on the MPSS, measured up to 4 weeks post-quit in abstinent and non-abstinent smokers across both trial arms.

• Prolonged abstinence measured and biochemically validated at 4 weeks post-quit and each follow-up using the Russell standard [39]. Criteria for the Russell standard includes a 2 week grace period from quit day, followed by smoking no more than 5 cigarettes and verification by means of exhaled CO, with a cut-off point of <10 ppm.

• Time to first lapse, with a lapse episode defined here as any smoking, even a puff [39].

### Other trial outcomes

• Feasibility of running the ARTS trial within NHS SSS assessed on the basis of:

• - Rates of response to patient invitation letters;

• - Rates of recruitment at telephone screening;

• - Rates of attendance at clinic visits;

• - Rates of drop out prior to and after randomisation.

• User acceptability as measured by ratings of perceived usefulness on a patient satisfaction questionnaire.

• Change in cue-induced cravings measured on the VAS prior to and at the end of the cue-exposure task at 4 weeks, 8 weeks, 3 months and 6 months post-quit day in abstinent and non-abstinent smokers across both trial arms.

• Measure of cognitive processing bias on the pictorial Stroop task, to assess generalisation of AR effects at 4 weeks post-quit in abstinent and non-abstinent smokers across both trial arms. Stroop bias will be measured by the difference in median reaction time taken to respond to colour-naming of smoking-related stimuli versus colour-naming of neutral stimuli.

• Measure of attentional bias towards novel untrained stimuli on the visual probe task at 4 weeks post-quit in abstinent and non-abstinent smokers across both trial arms.

• Measure of attentional bias on the visual probe task and pictorial Stroop task at 8 weeks, 3 months and 6 months to assess long term effects of AR.

• Strength of urge to smoke and withdrawal symptoms on the MPSS, measured up to 8 weeks, 3 months and 6 months to assess long term effects of AR.

### Power calculation

The sample size is based on the following. In these calculations, we assume that only quitters will continue to attend clinic and that the measures will be analysed primarily in abstinent smokers, as is standard practice with withdrawal phenomena [60].

We assume conservatively that the effect of 5 sessions of AR will be no greater than the effect of a single session. From the findings of the Attwood et al. study [26], to detect a mean reduction of 26 ms (SD = 43 ms) with 80% power and a type 1 error rate of 5%, 42 participants in each group will be required. We revised this calculation to adjust for baseline attentional bias scores. In our pilot study of AR, we found an estimated correlation coefficient of −0.13 between baseline and post-training measurements. Thus, to detect a reduction of 26 ms with the same standard deviation, power and type 1 error stated above, 42 participants are still required in each group. We expect that at least 50% of participants will reach the Russell standard abstinence criteria at 4 weeks, as the NHS services achieve greater than this, providing about 50 abstinent participants in each arm, sufficient to test this hypothesis.

The trial is an exploratory study but is powered to detect differences in urge to smoke. One study on smokers quitting on pharmacotherapy found that the mean change in urge strength between quit day and week 1 was about 0.5 points measured with the MPSS and had a standard deviation of 1.2 [61]. Another study reported that glucose reduced urge strength by 1.0 points, although this was immediately after dosing [62]. In both of these studies, MPSS urge strength was scored 0–5 [42]. We assume that if AR can reduce urge strength by 0.6 points, then 62 participants in each group will be needed to detect this with 80% power and a type 1 error rate of 5%. From the earlier study [61] we used an estimated correlation coefficient of 0.41 between quit day and post-training urge strength to adjust this power calculation. This means that to detect a 0.6 point reduction in urge strength (SD = 1.2) with 80% power and a type 1 error rate of 5%, 53 participants would be required in each group. In the first 4 weeks, when withdrawal is at its height, this implies that about 200 smokers will be needed, assuming that 60% will achieve abstinence in the first 4 weeks.

The trial is not large enough to detect the effects of AR on prolonged abstinence as several hundred participants would be needed. With a sample size of 200 smokers, if AR increased abstinence rates by 30% (RR = 1.3), we have approximately 57% power to detect a difference in the proportion abstinent, using a two-sided test with a type 1 error rate of 5% and assuming an abstinence rate of 50% in the control group.

### Loss to follow-up

Participants who fail to attend clinic and do not respond to our telephone calls will be classed as smokers for the analysis of smoking abstinence, as is standard [39]. We expect to make contact with more than 90% of people at 6 month follow-up, based on experiences of a recent trial [61]. We anticipate that the effects of AR on attentional bias and withdrawal phenomena will be analysed primarily in abstinent smokers, as recommended by Shiffman et al. (2004) [60], so defaulting from routine clinic appointments by failed quitters is not considered a threat on the integrity of the trial. We therefore do not require those participants who failed to maintain abstinence and abandoned their quit to continue to attend clinics except for reasons detailed below.

This study could give valuable information on what happens to attentional bias over time, how it is affected by training, how it is affected by resuming smoking, and whether the training effect is contingent on continued abstinence. Accordingly, we will ask all participants regardless of smoking status to attend the follow-up visits. Adequate compensation should increase the likelihood of attendance.

### Analysis

• Primary analyses

• Attentional bias scores on the visual probe task will be calculated by subtracting median RTs to probes that replace smoking-related pictures from median RTs to probes that replace neutral pictures, with positive scores indicating a bias towards smoking cues and negative scores indicating a bias towards neutral cues. Median RTs will be used because distributions of mean RTs are often reported as skewed [33,63]; therefore we do not need to set parameters for outlying RTs. Bias scores, as measured at 4 weeks post-quit, will be used to examine AR effects on attentional bias firstly by trial arm and secondly by abstinence status using ANCOVA. An alpha level of 0.05 will be used. These analyses will be performed using PASW Statistics 18 (SPSS, Inc., 2009, Chicago, IL, USA).

• To investigate AR effects on weekly urge to smoke, data will be analysed using a mixed effects regression model with an autoregressive variance-covariance structure, to allow for variations in craving between participants. This will enable all weekly time points to be included and modelled simultaneously. An autoregressive modelling structure takes into consideration that repeated craving measurements taken closer together in time on the same participant are likely to be more highly correlated than measurements that are taken further apart in time [64]. This modelling technique will be used for MPSS composite scores for urge to smoke (MPSS-C). Regression coefficients, p-values and 95% confidence intervals (CI) will be derived from the models. These analyses will be undertaken using Stata 12.0 (StataCorp, 2011, College Station, TX: StataCorp LP).

• Intention-to-treat analyses will be performed to account for people who drop out of treatment.

• Secondary analyses

• Effects of AR on withdrawal will be examined using the same technique stated for urge to smoke analyses, in a mixed effects regression model of composite scores for withdrawal symptoms (MPSS-M). We will control for baseline MPSS-M scores, as is standard for the MPSS [42].

• To determine the proportion of people achieving abstinence by trial arm, risk ratios (RRs) will be calculated with corresponding 95% CIs. Those reported as lost-to-follow up will be counted as non-abstinent, as is standard in the reporting of smoking cessation trials [39,65].

• Proportional hazards modelling will be used to analyse the median time to lapse by trial arm; hazard ratios (HRs) will be reported with corresponding 95% CIs. These analyses will be performed using Stata 12.0 (StataCorp, 2011, College Station, TX: StataCorp LP).

• Additional analyses

• We will adjust analyses of attentional bias scores, MPSS-C scores and MPSS-M scores for potential moderators of attentional bias, urge to smoke and withdrawal symptoms. These will include age, gender and FTND across all analyses. Pre-quit urge to smoke will also be examined in the model of attentional bias scores and pre-quit attentional bias in the models of MPSS-C and MPSS-M.

• To investigate longer-term retraining effects, we will analyse 8 week, 3 month and 6 month post-quit data for attentional bias scores using ANCOVA and mixed-effects regression models for MPSS-C scores and MPSS-M scores, as stated previously.

• To examine AR effects on cue-induced craving, VAS scores will be analysed in the same way as MPSS-C and MPSS-M scores, in mixed-effects regression models. Visual analogue scale scores will be calculated from measurements taken from a 0–100 mm scale before and after the cue exposure task, which are administered 1 week before quit day and again at 4 weeks, 8 weeks and 3 months post-quit. The difference between pre and post measurements will be calculated and the change in cue-induced craving over time will be reported.

• Generalisation of AR to other cognitive bias measures will be assessed using RT scores from the pictorial Stroop task. Stroop bias scores will be calculated by subtracting median RTs to probes that replace neutral pictures from median RTs to probes that replace smoking-related pictures. Slower RTs towards smoking-related pictures indicate a bias towards smoking cues. Again, parameters for outlying RTs do not need to be defined as median rather than mean RTs will be used. Bias scores, as measured at 4 weeks post-quit, will be used to examine AR effects on Stroop bias firstly by trial arm and secondly by abstinence status using ANCOVA.

• Similarly, we will assess whether AR generalises to untrained novel stimuli that only appear in the assessment versions of the visual probe task and not during training sessions. Attentional bias scores for the trained and untrained stimuli will therefore be analysed separately using ANCOVAs.

• Responses to the patient satisfaction questionnaire and identification of group allocation will be compared across trial arms as percentages.

### Ethics and research governance

The trial will be conducted in compliance with the principles of the Declaration of Helsinki (1996), the ICH-GCP, the EU Clinical Trials Directive and all applicable regulatory requirements. The study protocol and other documentation were approved by the National Research Ethics Committee (10/H1206/34) and local NHS Research & Development offices. Subsequent protocol amendments will be submitted to the Research Ethics Committee for approval, and the other bodies where necessary. We will provide the Research Ethics Committee with annual progress reports, in addition to a final study report.

## Discussion

This is the first trial to assess the potential clinical translation of AR procedures in smokers attempting to quit using NHS SSS. Multiple sessions of AR might increase treatment efficacy on top of standard treatment, as demonstrated in a recent trial of alcohol-dependent patients [33]. If effective, not only could AR be used in stop smoking clinics, it could also be offered as a web-based intervention on NHS stop smoking websites as another potentially low-cost alternative.

Several procedures are in place to minimise potential sources of bias. To minimise the risk of selection bias, we are using a simple random sequence for assigning participants to groups. To minimise performance bias, we are blinding all therapists and participants to group allocation, though there is a risk of participants becoming aware of which group they have been assigned to while carrying out the task itself. At follow up, we will assess participants’ knowledge of which intervention they believe to have received.

While this trial offers the ability to examine AR as a therapeutic tool, data on attentional bias and its relation to urges to smoke, cue-induced cravings and resumption of smoking will enhance our understanding of relapse and possible preventative strategies. Knowing how urges to smoke strike, their antecedents and smoker’s responses to these are a key part of designing future intervention strategies.

## Competing interests

RB, LN, MAM, RH, SS, SSu and SGF have no competing interests. PA has done research and consultancy for manufacturers of smoking cessation medication.

## Authors’ contributions

MRM and PA conceived the study. RB, PA, MRM, SS, RH and SSu were involved in the initial discussions that led to the grant application and writing of the study protocol. RB, PA, MRM, SS, SGF and SSu participated in the study design. RH is the trial statistician and together with LN, MAM, PA and RB devised the statistical analysis plan. All authors contributed to the draft of the manuscript. All authors read and approved the final version of the manuscript.

## Pre-publication history

The pre-publication history for this paper can be accessed here:

http://www.biomedcentral.com/1471-2458/13/1176/prepub
